# Dengue Virus Type 4 Phylogenetics in Brazil 2011: Looking beyond the Veil

**DOI:** 10.1371/journal.pntd.0001439

**Published:** 2011-12-27

**Authors:** Renato Pereira de Souza, Iray M. Rocco, Adriana Y. Maeda, Carine Spenassatto, Ivani Bisordi, Akemi Suzuki, Vivian R. Silveira, Sarai J. S. Silva, Roberta M. Azevedo, Fernanda M. Tolentino, Jaqueline C. Assis, Margarida G. Bassi, Bibiana P. Dambrós, Gabriela L. Tumioto, Tatiana S. Gregianini, Luiza Terezinha M. Souza, Maria do Carmo S. T. Timenetsky, Cecília L. S. Santos

**Affiliations:** 1 Centro de Virologia, Instituto Adolfo Lutz, São Paulo, Brazil; 2 Núcleo de Ciências Biomédicas, CLR - Instituto Adolfo Lutz de São José do Rio Preto, São José do Rio Preto, Brazil; 3 Seção de Virologia, Laboratório Central de Saúde Pública do Estado do Rio Grande do Sul - Fundação Estadual de Produção e Pesquisa em Saúde, Porto Alegre, Brazil; Centers for Disease Control and Prevention, United States of America

## Abstract

Dengue Fever and Dengue Hemorrhagic Fever are diseases affecting approximately 100 million people/year and are a major concern in developing countries. In the present study, the phylogenetic relationship of six strains of the first autochthonous cases of DENV-4 infection occurred in Sao Paulo State, Parana State and Rio Grande do Sul State, Brazil, 2011 were studied. Nucleotide sequences of the envelope gene were determined and compared with sequences representative of the genotypes I, II, III and Sylvatic for DEN4 retrieved from GenBank. We employed a Bayesian phylogenetic approach to reconstruct the phylogenetic relationships of Brazilian DENV-4 and we estimated evolutionary rates and dates of divergence for DENV-4 found in Brazil in 2011. All samples sequenced in this study were located in Genotype II. The studied strains are monophyletic and our data suggest that they have been evolving separately for at least 4 to 6 years. Our data suggest that the virus might have been present in the region for some time, without being noticed by Health Surveillance Services due to a low level of circulation and a higher prevalence of DENV-1 and DENV- 2.

## Author Summary

Dengue virus infections are a major concern in developing countries, affecting approximately 100 million people/year. The virus has four immunologically related serotypes (DENV-1, DENV-2, DENV-3 and DENV-4) associated with human disease. The virus is widespread in tropical and Sub-Tropical areas of Asia, Africa and Americas. The virus is transmitted by mosquito bites, and is primarily associated with *Aedes aegypti* as its main vector. To understand the reemergence of DENV-4 in Brazil in 2010–2011 we carried out a Bayesian phylogenetic analysis of the envelope gene sequences sampled in Brazil in 2011. Our results indicate that the studied samples are close related to strains circulating since 1981, when DENV-4 was first introduced in South America, but have gone trough recent evolution for at least 4 to 6 years. Our results also suggests that the virus may have penetrated Brazilian population earlier than 2010, indicating that the virus could have been present but not detected due a higher prevalence of DENV-1 and DENV- 2 and the failure of the surveillance system to locate the milder disease commonly associated with DENV-4.

## Introduction

Dengue virus (DENV) is a single stranded RNA virus, with four immunologically related serotypes (DENV-1, DENV-2, DENV-3 and DENV-4) associated with Dengue Fever (DF) and Dengue Hemorrhagic Fever (DHF) [1].

The virus is widespread in tropical and Sub-Tropical areas of Asia, Africa and Americas. The virus is transmitted by mosquito bites, and is primarily associated with *Aedes aegypti* as its main vector [2].

The disease affects, approximately, 100 million people/year, causing 250,000 cases of DHF with a case fatality rate up to 15%, and is a major concern for Public Health authorities around the globe, primarily in developing countries [2].

Historically, the State of Sao Paulo, Brazil, has been suffering dengue outbreaks since 1990 when DENV-1 was introduced in the area. Subsequent epidemics were detected in 1997 and 2002, caused by DENV-2 and DENV-3, respectively, with increasing casuistic and detection of severe cases of DHF or Shock Syndrome [3]–[5].

DENV-4 had a brief circulation in Brazil in 1982 in the Northwestern region of Brazilian Amazon in a focal epidemic. No further cases of infection had been registered in the country until 2008, when the virus was detected in three patients, who had no international traveling history, in Manaus [6].

After this episode, the Brazilian Ministry of Health implemented the use of the NS1 ELISA test in 16 states in order to increase the percentage of viral isolates and the determination of the serotypes circulating in the country. Before the screening with the NS1 ELISA test, virus isolation was obtained in only 10% of samples submitted to isolation. With the screening of samples the percentage of detection of serotype rose to 82% [7]. The introduction of the NS1 ELISA assay as a tool for screening positive samples led to an important increase in the success of virus isolation. In São Paulo State, only 33.3% of the total of the samples inoculated in 2008 resulted in successful virus isolation, while in 2009 and 2010, 85.7% succeeded. The number of São Paulo state counties that sent samples for isolation also increased from 0.9% in 2008 to 10.2% in 2009 (Bisordi I, 2011, unpublished data).

DENV-4 reemerged in the country in 2010 in the municipalities of Boa Vista and Cantá in Roraima State [8]. The virus spread to different geographic regions of Brazil with cases of infection registered in the North (Roraima, Amazonas, Pará), Northeast (Bahia, Pernambuco, Piauí) and Southeast (Rio de Janeiro, Sao Paulo) [9].

Despite the importance of the virus distribution, little is known about its rate, pattern of spreading and evolution. Each serotype represents a cluster of different genetic lineages constantly evolving and changing within the population [10].

In the present work, six strains of the first autochthonous cases of DENV 4 infection occurred in Sao Paulo State and Rio Grande do Sul State, Brazil, in 2011 were studied using a Bayesian Phylogenetic approach. Nucleotide sequences of the envelope gene were determined and compared with the corresponding sequences of representative strains of the known DENV-4 genotypes. The main objectives of the present study are the identification of the genotypes of the newly introduced strains, the examination of the phylogenetic relationships between strains and the estimation of emergence time of DENV-4 strains.

## Methods

### Ethics Statement

The specimens analysed in this study were retrieved from a collection formed from materials received for diagnostic purposes in the Instituto Adolfo Lutz. The samples were sent by reference hospitals and the patients names are confidentially anonymized, and only reference numbers were used during the diagnostic procedures and in the analysis that originated this study.

### Virus

All new DENV-4 strains characterized in this study were isolated directly from patient serum and detected by RT-PCR between February and March of 2011. The origin of the strains are detailed in Table 1.

**Table 1:** DENV-4 virus strains included in phylogenetic analysis of envelope gene.

Strain ID	Location	Dating (Year)	Access Number
DENV-4/BB/12102/1993	Barbados	1993	AY152375
DENV-4/BB/9312112/1993	Barbados	1993	AY152376
DENV-4/BB/9908743/1999	Barbados	1999	AY152368
DENV-4/BR/1385/1982	Brazil	1982	U18425
DENV-4/BS/9809160/1998	Bahamas	1998	AY152366
DENV-4/CN/CN78-56/1978	China	1978	EF436279
DENV-4/CN/D10166-GZ/2010	China	2010	JN029828
DENV-4/CN/GD09/1990	China	1990	FJ196850
DENV-4/CO/371813/1996	Colombia	1996	DQ341219
DENV-4/CO/BID-V3409/2001	Colombia	2001	GQ868582
DENV-4/CO/BID-V3410/2004	Colombia	2004	GQ868583
DENV-4/CO/BID-V3412/2005	Colombia	2005	GQ868585
DENV-4/CR/108/1996	Costa Rica	1996	AH011968
DENV-4/DM/814669/1981	Dominica	1981	AF326573
DENV-4/DM/M.44/1981	Dominica	1981	AY152360
DENV-4/GP/FWI/2004	Guadeloupe	2004	DQ390320
DENV-4/HN/F07-076/2007	Honduras	2007	GU586124
DENV-4/HN/HON_1991/1991	Honduras	1991	AY152379
DENV-4/ID/0712aTw/2007	Indonesia	2007	EU448463
DENV-4/ID/1036/1976	Indonesia	1976	U18429
DENV-4/ID/1132/1977	Indonesia	1977	U18430
DENV-4/ID/30153/1973	Indonesia	1973	U18428
DENV-4/ID/SW36i/1984	Indonesia	1984	AY858049
DENV-4/ID/SW36i/2004	Indonesia	2004	AY858049
DENV-4/ID/SW38i/2004	Indonesia	2004	AY858050
DENV-4/IN/ND-73/2007	India	2007	HM237348
DENV-4/JM/0886/1983	Jamaica	1983	AY152384
DENV-4/JM/1082/1981	Jamaica	1981	AY152389
DENV-4/JP/61NIID/1961	Japan	1961	AB111090
DENV-4/KH/0509aTw/2005	Cambodia	2005	EU448455
DENV-4/LK/17/1978	Sri Lanka	1978	AY550909
DENV-4/MQ/FWI/2004	Martinique	2004	DQ390319
DENV-4/MS/9412570/1994	Montserrat	1994	AY152371
DENV-4/MX/111/1995	Mexico	1995	AH012018
DENV-4/MX/1420/1983	Mexico	1983	DQ341211
DENV-4/MX/1492/1984	Mexico	1984	U18431
DENV-4/MX/1551/1985	Mexico	1985	DQ341213
DENV-4/MX/1554/1985	Mexico	1985	DQ341214
DENV-4/MX/4959/1995	Mexico	1995	DQ341216
DENV-4/MX/6637/1997	Mexico	1997	DQ341218
DENV-4/MX/Cardenas-2/2006	Mexico	2006	HM171571
DENV-4/MY/H64/2006	Myanmar	2006	EU478408
DENV-4/MY/P7-1006/1969	Malaysia	1969	AF231722
DENV-4/MY/P73-1120/1973	Malaysia	1973	AF231724
DENV-4/MY/P75-215/1975	Malaysia	1975	EF457906
DENV-4/MY/P75-514/1975	Malaysia	1975	AF231723
DENV-4/NC/5489/1984	New Caledonia	1984	DVU18432
DENV-4/PE/FST1425/2008	Peru	2008	GQ139560
DENV-4/PE/OBT1158/2000	Peru	2000	GQ139564
DENV-4/PE/SER6269/2007	Peru	2007	GQ139562
DENV-4/PF/114094/1985	French Polynesia	1985	U18439
DENV-4/PF/S-44754/1979	French Polynesia	1979	U18438
DENV-4/PH/0409aTw/2004	Phillipines	2004	EU448458
DENV-4/PH/H241/1956	Phillipines	1956	AB609591
DENV-4/PR/1650/1986	Puerto Rico	1986	U18436
DENV-4/PR/20/1998	Puerto Rico	1998	AH011951
DENV-4/PR/63/1987	Puerto Rico	1987	AH012006
DENV-4/PR/96/1990	Puerto Rico	1990	AY152855
DENV-4/PR/M.20/1982	Puerto Rico	1982	AH012031
DENV-4/PR/M33/1985	Puerto Rico	1985	AY152857
DENV-4/SB/0712aTw/2007	Solomon Islands	2007	EU448462
DENV-4/SG/0108aTw/2001	Singapore	2001	EU448464
DENV-4/SG/06K2270DK1/2005	Singapore	2005	GQ398256
DENV-4/SG/2641Y08/2008	Singapore	2008	HQ875339
DENV-4/SR/114217/1994	Suriname	1994	AY152373
DENV-4/SR/824188/1982	Suriname	1982	AY152388
DENV-4/TH/0017/1997	Thailand	1997	AY618989
DENV-4/TH/0034/1994	Thailand	1994	AY618972
DENV-4/TH/0087/1977	Thailand	1977	AY618991
DENV-4/TH/0100/1995	Thailand	1995	AY618974
DENV-4/TH/0104/1986	Thailand	1986	AY618962
DENV-4/TH/0164/1999	Thailand	1999	AY618986
DENV-4/TH/0229/1996	Thailand	1996	AY618977
DENV-4/TH/0348/1991	Thailand	1991	AY618990
DENV-4/TH/0358/1992	Thailand	1992	AY618968
DENV-4/TH/0417/1984	Thailand	1984	AY618959
DENV-4/TH/0476/1997	Thailand	1997	AY618988
DENV-4/TH/0485/1995	Thailand	1995	AY618975
DENV-4/TH/0485/2001	Thailand	2001	AY618992
DENV-4/TH/0521/1999	Thailand	1999	AY618987
DENV-4/TH/0557/1991	Thailand	1991	AY618966
DENV-4/TH/0734/2000	Thailand	2000	AY618993
DENV-4/TH/1270/1998	Thailand	1998	AY618981
DENV-4/TH/182/1985	Thailand	1985	AY618961
DENV-4/TL/ET00/2000	East Timor	2000	AY705988
DENV-4/TT/841223/1984	Trinidad and Tobago	1984	AY152381
DENV-4/TT/9908820/1999	Trinidad and Tobago	1999	AY152367
DENV-4/TT/TPHL4233/1982	Trinidad and Tobago	1982	AY152383
DENV-4/US/BID-V1082/1998	USA	1998	FJ024424
DENV-4/US/BID-V1083/1986	USA	1986	EU854295
DENV-4/US/BID-V2431/1995	USA	1995	GQ199880
DENV-4/US/BID-V2435/1996	USA	1996	GQ199881
DENV-4/US/BID-V2448/1999	USA	1999	FJ882601
DENV-4/US/BID-V860/1994	USA	1994	FJ226067
DENV-4/VE/113/1995	Venezuela	1995	AH011965
DENV-4/VE/24082/2004	Venezuela	2004	GQ139588
DENV-4/VE/29056/2005	Venezuela	2005	GQ139590
DENV-4/VE/39504/2007	Venezuela	2007	GQ139591
DENV-4/VE/8616/2001	Venezuela	2001	GQ139586
DENV-4/VE/BID-V1153/2007	Venezuela	2007	GQ868642
DENV-4/VE/BID-V1154/2007	Venezuela	2007	GQ868643
DENV-4/VE/BID-V1155/2007	Venezuela	2007	GQ868644
DENV-4/VE/BID-V1156/2007	Venezuela	2007	GQ868645
DENV-4/VT/382VN/2001	Viet Nam	2001	AY786201
DENV-4/VT/480VN/2002	Viet Nam	2002	AY786202
DENV-4/VT/TG879/1990	Viet Nam	1990	AY786200
DENV-4/VT/TN2693/1999	Viet Nam	1999	AY786198
DENV-4/BR/SPH 317947/2011*	Brazil	2011	JN092553
DENV-4/BR/SPH323844/2011*	Brazil	2011	JN848496
DENV-4/BR/SIGH20011019044/2011*	Brazil	2011	JN848497
DENV-4/BR/SPH319325/2011*	Brazil	2011	JN848498
DENV-4/BR/SPH/318527/2011*	Brazil	2011	JN848499
DENV-4/BR/SPH320649/2011*	Brazil	2011	JN848500

* this study.

### Virus isolation in cell culture

Twenty microliters of the patients blood or serum were inoculated in tubes seeded with cultured cells of *Aedes albopictus*, clone C6/36. Indirect immunofluorescence assay (IFA) with polyclonal anti-flavivirus antibodies and anti-mouse immunoglobulin conjugated (fluorescein isothiocyanate – Sigma) were performed [11]. The positive samples were typed by IFA with monoclonal antibodies to DENV (Biomanguinhos).

### RNA extraction and RT-PCR

Total RNA was extracted from the supernatant fluid of C6/36 infected cells using the commercial kit QIAamp® Viral RNA (Qiagen Inc., Ontario, CA), according to the manufacturer's instructions.

One step RT-PCR was performed employing the protocol described by Lanciotti et al, [12] in the presence of a set of primers targeting the complete envelope gene sequence, described by Lanciotti *et al* [13]. RT-PCR products were purified and directly sequenced using the Big Dye v.3.1 terminator chemistry. Sequences were determined using the Applied Biosystems 3130XL DNA sequencer.

All nucleotide sequences of the envelope gene for DENV-4 serotype generated for this study are deposited in GenBank under accession numbers JN092553 and JN848496–JN848500 (Table 1).

### Phylogenetic Analysis

Sequences representative of the known genotypes I, II, III and Sylvatic for DEN4 were retrieved from GenBank and included in the phylogenetic analysis for comparison with the sequences generated in this study (Table 1). Sequence alignment was performed using the BioEdit software [14].

The Bayesian inference method available in the software BEAST v. 1.6.2 was used in order to analyze the phylogenetic relationship of the strains of this study [15]. The analysis of phylogenetic relationships and evolution, encompassed the entire Envelope gene, including six DENV-4 strains generated in this study and 107 sequences retrieved from GenBank (Table 1).

Each sequence of the corresponding data set was dated and maximum clade credibility (MCC) tree was generated. The internal nodes were inferred using a Markov Chain Monte Carlo (MCMC) Bayesian approach under a GTR model with Gamma-distributed rate variation (*γ*) and a proportion of invariable sites (I), using a relaxed (uncorrelated lognormal) molecular clock. Previously published data [10], [16] suggest that dengue evolution generally approximates a molecular clock with occurrence of minor differences in rate. Four independent MCMC runs of four chains each were run for 10 millions generations. Convergence of parameters during MCMC runs were assessed by their Effective Sample Size (ESS) reaching values above 150 as calculated with Tracer V 1.5 [15]. We used a Bayesian skyline coalescent prior to estimating population dynamics through time and access an estimative of evolutionary rate and the time of the most recent common ancestor (TMRCA) in the Envelope gene analysis.

## Results

A fragment of 1487 nucleotides representing the entire sequences encoding the envelope gene was determined from 6 strains of DENV-4 and further aligned with other 107 envelope gene sequences retrieved from GenBank.

**Figure 1:**
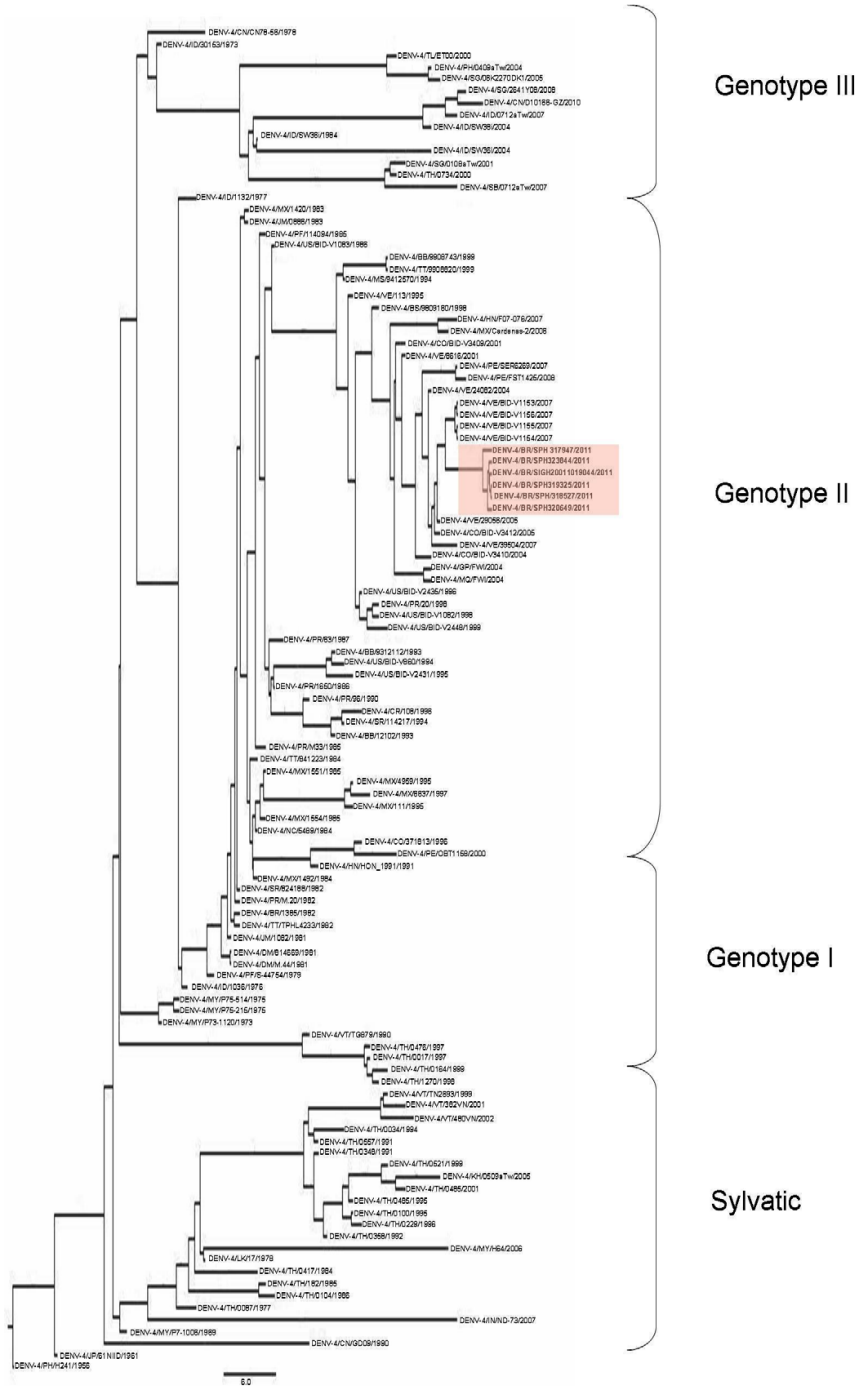
Envelope gene MCMC tree. The internal nodes were inferred using a Markov Chain Monte Carlo (MCMC) Bayesian approach under a GTR model with Gamma-distributed rate variation (*γ*) and a proportion of invariable sites (I), using a relaxed (uncorrelated lognormal) molecular clock. Four independent MCMC runs of four chains each were run for 10 millions generations. The highlighted sector indicates the position of the studied strains.

The phylogenetic relationships among those strains were reconstructed by Bayesian analysis with a relaxed (uncorrelated lognormal) molecular clock model. The analysis generated a MCMC phylogenetic trees (Fig. 1). All samples sequenced in this study were located in Genotype II, and coupled with samples from the Caribbean region and northern South America (Fig. 1). In general, the group is strongly supported (posterior probability of 0.98) with Internal relations within the clade showing a lower support, most likely due the higher homology of the samples, which hinders the separation, but the isolated strains are monophyletic in origin, supported by a high posterior probability (0,99).

The isolated strains in this study are monophyletic and our data suggest that they have been evolving separately for at least 4 to 6 years. Nonetheless, they are quite similar and relatively unchanged in relation to the DENV-4 introduced originally in the Caribbean region and northern South America.

The relaxed molecular clock estimated after the analysis of the envelope gene encopassed a time of evolution for DENV-4 of 50–60 years and an average replacement rate of 2.0037×10^−3^ Subs/Site/Year, considering an Effective Sample Size of 334.79 calculated in Tracer 1.5. The replacement rate of the branch of the isolated strains is of 1.238×10^−3^ Subs/Site/Year, and the branch originated within 4 to 6 years probably diverging from virus circulating in Venezuela as the closest sister branch reunited Venezuelan strains supported by a posterior probability of 0,99.

## Discussion

All sequenced strains were encompassed in genotype II, with a high medium posterior probability (0.98), slightly lower in the terminal clades due to the genetic similarity of samples which hinders the separation. The isolated strains formed a strongly supported monophyletic branch (posterior probability of 0,99).

Not all Brazilian samples included in this study belonged to genotype II. The sequence AM 1619, from Manaus, 2008, retrieved from GenBank, grouped with genotype I. Our data also support the recent circulation of DENV-4, genotype I, reported in Manaus County in 2008 [6].

The studied period of evolution of DENV-4 after the analysis of the Envelope gene was estimated between 50–60 years, with an average replacement rate of 2.0037×10^−3^ Subs/Site/Year, considering an Effective Sample Size of 334.79. This estimative is supported by previously published data [10], [17]. Our result strongly suggests that the introduction of genotype II in South America occurred between 30–35 years ago, most probably through the Caribbean region or the northern South America. These results corroborate previously published data, since the first cases associated with DENV-4 from the American Continent are dated around 1982, in the Caribbean islands [10], [13], [18].

These data indicate that Dengue evolution approximates a molecular clock with minor rating variances. It is interesting to observe that raising ratings are mostly associated with increasing case occurrences or the emergence of the virus in a new region, meaning that the virus, when confronted with a susceptible population, undergoes an explosion of diversity.

**Figure 2:**
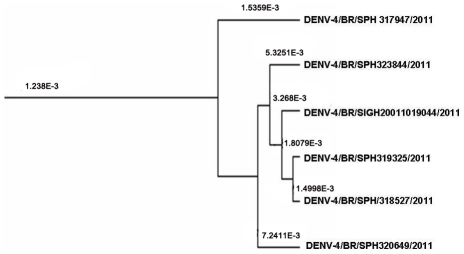
Detail of the Envelope gene MCC tree. Bolded numbers above branchs are the estimated replacement rates for each sample. The acceleration observed in this terminal branch ma be indicative of a recent introduction of the virus in a previously naïve population.

These phenomena were previously reported concerning Dengue and other Flaviviruses [1], [19]–[21]. The clade directly associated with the studied strains showed a replacement rate of 1.238×10^−3^ Subs/Site/Year, slightly under the average rate. However, the rates observed within the clade formed by the isolated strains show higher replacement rates when compared with the sister branches (Figure 2). Such findings may indicate that the virus started to evolve more quickly, suggesting that it may have recently found a susceptible population and is spreading.

The DENV-4 samples, sequenced in this study, represent a recent emergence of a viral strain circulating in South America around 20 to 25 years ago. Results suggest that a local evolution has been taking place for about 4 to 6 years. These data could indicate that the virus might have been present in the region for some time, without being noticed by Health Surveillance Services due to a low level of circulation and a higher prevalence of DENV-1 and DENV- 2. It is possible that, since DENV-4 is associated with a milder disease [22], [23], the human cases may have been below the line of screening, going unnoticed. It is probable that the recent efforts to increase the success of virus isolation and serotyping allowed the study of a greater number of cases that otherwise would not have been serotyped, enabling the notification of less prevalent serotypes.

However, the hypothesis of a recent introduction cannot be ruled out, but it would imply in multiple recent introductions of the virus, in a very short period of time, in relatively distinct areas, or a single introduction event in a significantly important area that facilitated the virus introduction in new areas. The simultaneously occurrence of DENV-4 in different Brazilian States, forming a strongly supported clade, in the beginning of 2011, favors a recent emergence of the virus followed by a quickly introduction. However, such occurrence did not provide any clue to substantiate whether the virus was widespread but circulating in a low level, or circulating in a restricted area and subsequently taken to new localities with susceptible hosts.

The isolated strains are monophyletic in origin and the molecular clock supports a local evolution, but by no means it indicates where that evolution occurred. It may have occurred in northern Brazil, and the virus quickly were introduced in Southern region due the constant human traffic. As the closest branch in our phylogenetic analysis is formed by Venezuelan strains of DENV-4, a Venezuelan origin of Brazilian DENV-4 may be a plausible hypothesis.

Either way, the virus may have evolved in an imperceptible manner in an undisclosed place, it was not reported and later emerged subtly and spread fast among a susceptible population. The recent DENV-4 cases reported elsewhere may represent a cryptic circulation that was only recently detected. The analysis of more sequences from a broader geographical perspective, encompassing other Brazilian regions, is crucial in order to understand how the virus evolved and how it got widespread.

The reemergence of DENV-4 should be a concern for Health authorities since there are evidences that the replacement of a dominant circulating genotype is associated with the rising of a previously rare lineage. These phenomena were observed in Puerto Rico [24] and could be a plausible scenario in Brazil.

The authors indicate the necessity to study the phylodynamics of Dengue virus and the dynamics of genotypes and serotypes circulation and substitution in the population.

It is equally necessary to extend the efforts of virus isolation and sequencing towards the mosquito population. The mosquitoes are a reliable source of information on circulating virus, as mosquitoes do not depend on medical screening or the spontaneous search for medical services by the symptomatic patients.

Our results indicate the recent circulation of DENV-4 in São Paulo.
